# Female Physicians

**Published:** 1861-10

**Authors:** 


### Art. IV.—FEMALE PHYSICIANS.

Why should woman be excluded from the legitimate practice of Physic ? why should she be debarred from wielding pill and potion ?—that is to say, according to the rules of art, for it is a painful fact that she is not excluded, neither does she exclude

---

herself from wielding them according to her own good will and pleasure. Who among the affectionate cares of nurse or mother, by which his childhood was fenced in, has not to enumerate the periodical administration of some nauseous domestic remedy? Young Hopeful is accustomed to awake with the lark, and he has acquired a happy faculty of arousing the whole house soon after he is astir. "My dear," testily exclaims the disturbed father to his equally disturbed (but not testy) wife, "I wish you would teach nurse to keep Johnny quieter until breakfast time." But this morning the mother sleeps long beyond the accustomed time of being aroused, and suddenly awaking she hears no noise but the melodious breathing of her spouse, slumbering quietly by her side, and the subdued sounds made by the housemaids in the lower parts of the house. Startled by this boding absence of the usual racket, she hastily puts on her dressing-gown, and hurries to the nursery. There she finds young Hopeful in bed, not asleep, but by no means vivacious. "What ails my dear boy?" cries the anxious mother, taking him in her arms. "Is he poorly?" Johnny responds to his mother's embrace by a half sulky caress, and the nurse interfering, proceeds to explain that he has had a somewhat restless night (meaning that he awoke once before his usual time), and that she thinks he is a little feverish. Then mother and nurse, putting their heads together, and having in mind certain indulgences in the shape of sweetmeats which young Hopeful had been permitted to revel in the day previous, come to the conclusion that it would be prudent to give him, by way of corrective, a little of that time-honoured combination and chiefest of all domestic remedies—Senna and Salts. The nauseous draught is ready, and nurse taking Johnny on her knee, advances it to his mouth; but no sooner does the odour reach his nostrils, than he starts back with a loud cry. Nurse and mother in vain endeavour to persuade the child to swallow the sickly stuff. Tossing about, kicking, and shrieking, he sets the best intentioned efforts at defiance. Sixpences and shillings tempt not, sweetmeats he repudiates with scorn, and promises of marvellous toys fall unheeded upon his ears. The nurse begins to be vexed and imperative, and the mother looks distressed. The draught must be taken. The necessity is awful, but absolute. Both nurse and mother recognise the terrible struggle now involved in the administration. The nurse would, however, terminate the contention at once by main force, but the mother still hopes to succeed by gentler means. "Johnny, you *must* take the physic," exclaims the nurse, with a significant shake of the child. "I wont," shrieks Johnny. "Don't hurt him, nurse," cries the mother, the tears forming in her eyes. "Johnny dear, wont you take it to please Ma?" "No-o-o!" blubbers Johnny.

"You naughty boy," says nurse. "I must send for your father," cries the mother. And in the end the father is called from his dressing-room, having been duly impressed with the gravity of the occasion. With a big voice, but a faint heart, he confronts the recalcitrant child, cup in hand. "Johnny, you will take this from me." "No," retorts Johnny, with a kick and a sob. "Then I shall whip you, Johnny;" but the threat makes no impression. "What must be done?" then asks the nonplussed father, now utterly helpless. "Make him take it," answers the nurse, in a peremptory tone; "what's the use of trifling with him in this way?" A housemaid is called up to aid. The nurse throws Johnny on his back over her knees, he screaming loudly. The housemaid holds him tightly by the arms and shoulders, the mother secures the legs, the father steadies the body. Then the nurse, seizing the nose of the child between the thumb and finger of the left hand, compresses his nostrils until he opens his mouth widely, whereupon she pours into it the abominable draught. Part is swallowed, but the greater part is sputtered out, bespattering the housemaid's neat print dress, and the nurse's face. The nurse is put out of temper for the day on account of the naughtiness of the child; the mother is vexed that he has had to be dealt with so severely; the housemaid is annoyed because her clean dress is spoiled; the father is tetchy because his matutinal bloater happening to be ready for him before he was ready for it, has become lukewarm; and the cook is indignant, because he was cross with her needlessly. And all this turmoil in the internal economy of the house is but a faint reflex of the turmoil in the unfortunate Johnny's internal economy.

In our own boyish days senna and Epsom salts, and treacle and brimstone (horrific compound!) formed part of the "institutions" of childhood. No household would have been considered well ordered which did not contain among its permanent stores the drugs we have mentioned, together with rhubarb, magnesia, and sundry "simples;" no housewife would have been regarded as at all equal to her duties who was not versed in "Buchan," and prepared at any moment to dose any of her household upon the slightest occasion.* The passion for physicking among housewives is not a whit less marked now than then. In certain districts

* "Old Gervase Markham, in his 'Approved Book called the English Housewife, containing the inward and outward virtues which ought to be in a complete woman,' places her skill in physic as one of the most principal. 'You shall understand,' he says, 'that sith the preservation and care of the family touching their health and soundness of body consisteth most in her diligence, it is meet that she have a physical kind of knowledge, how to administer any wholesome receipts or medicines for the good of their healths, as well to prevent the first occasion of sickness, as to take away the effects and evil of the same, when it hath made seizure upon the body.' And, 'as it must be confessed that the depths and secrets of this more excellent art of physic are far beyond it may have assumed a more refined and less aggressive character, but its vitality is not on that account diminished. The much-revered "Buchan" has been supplanted by a host of other (and better) works, simplifying medicine to the capacity of the domestic mind. The cleverest thing that homœopathy ever did in a worldly-minded point of view, was to pander to the disposition existing among ladies for domestic physic. What chance had the sickly senna, the bulky magnesia, the nauseous salts and the gritty rhubarb against the elegant materia medica, complete in itself, packed within the compass of a lady's reticule, and of which the drugs were tasteless, odourless, and not offensive to the sight? But idealism in physic seldom holds extensive sway long. However much people may abhor drugs in their unsophisticated state, they quickly lose faith in them when they are so disguised as to be deprived of all sensible qualities. Among the uneducated in many parts of the country it is an article of belief that the beneficial effects of a drug are in proportion to its nastiness of taste. "I reckon nought of physic that's gotten no taste in 't," we heard one of the illiterate say not long ago. The word "physic," in fact, has come to have a specific secondary signification attached to it, implying nauseousness of taste or smell, as when we say a thing is "physicky." This highly sensualistic view has a much wider and more extended influence over people than might be at first imagined, and it has exercised an important influence in checking and controlling, and ultimately subduing the rage which at one time prevailed for homœopathic domestic medicine. The refined charlatanry was no match against the rough common sense which is happily tolerably plentiful among us; and it only found a lodgment among those who cared to exercise their imagination rather than their ratiocinative powers, and who were too highly educated to pay heed to the very vulgar dictates of ordinary reason.

The doctor is often sadly pestered by the passion for giving physic among ladies. No sooner has he thrown light upon the nature of the case he has been called to, than some work on domestic medicine is consulted by the lady or ladies of the house, and, unless he has great tact in putting an immediate end to the nuisance, he is apt to be sadly troubled with suggestions or questions as to why he did not do this, that, or the other thing. Frequently the

---

the capacity of the most skilful woman,' he relates for the housewife's use some 'approved medicines and old doctrines gathered together by two excellent and famous physicians, and in a manuscript given to a great worthy Countess of this land.' And the author of '*the English Gentleman and the English Gentlewoman*,' presented to present times for ornaments, and commended to posterity for precedents, says of the gentlewoman, 'herbals she peruseth, which she seconds with conference; and by degrees so improves her knowledge, as her cautelous care perfits many a dangerous cure."—See Southey's *Doctor*, pp. 57, 59.

annoyance takes another form. "Doctor, Mrs. So-and-so tells me that when her child was suffering in this manner, Doctor So-and-so ordered so-and-so. Would it be proper to do the same in this case?" Not long ago a distinguished member of the Medical Council was giving most careful instructions to the nurse in an exceedingly grave case, when one of the ladies of the family who was present, interfered and suggested the addition of a domestic diet-drink of local celebrity to the general treatment of the case. "Madam," was the sharp and vexed retort of the doctor, "if you wish to take charge of the case, I shall be most happy to give place to you."

It is in this, we may almost call it instinctive propensity of woman to deal with remedies, that we find the best explanation of that sometimes agitated and most recondite problem, the primitive origin of medical art. Now, Plato* long ago intimated that if woman had a talent for physic she was as well fitted as man to cultivate it. The evidence that she possesses such a talent is surely very strong. Why, then, is she excluded from the legitimate practice of the art?

It would naturally be thought that her delicate and taper fingers were more fitted to gauge the pulse than man's; that her eyes would at least be as sharp as his in detecting a morbid sign, her ears as quick in catching a morbid sound, her ready wit more apt to analyse symptoms, and her readier sympathy to secure the confidence of the patient.

We must confess, for our own part, to a slight weakness in favour of lady-doctors. It is, perhaps, a faded but still pleasant reminiscence of youthful days. Whatever objection might at first have been experienced by us to the administration of domestic remedies, it was quickly modified when the true representative of Æsculapius had to come into the field. The domestic physic, although bulky and disgusting, was still as a rule but a single administration, and usually led to a little after-petting, in which the palate came off most happily. But the doctor's physic, equally offensive, although less bulky, had to be taken with awful regularity at stated intervals; and, adding insult to injury, he would pat our head in the kindest manner, while in the blandest tones, he would quietly put aside the anxious suggestions of mother and nurse, that we might have this or that little knick-knack. "No, no, my dear Madam," he would say, his face creaming with a pleasant smile: "No, no, Nurse, wait a little longer. A little gruel or weak broth is all Johnny requires at present."

Ah! how pleasant it was to read of the doughty Sir Bevis of Hampton, tenderly bephysicked by the lovely Josyan. To hear

---

* *Republic*, B. v. § 5.

in imagination the nascent hero exclaiming, when as a page he had contrived to kill in one solitary tussle sixty pagan knights, and after a few preliminary affectionate passages, "O Josyan, of thy grace aid me, for I am sorely wounded." "Sweet leman," answered she, "I am a leech equal with the best. No salve exists in all Pagandom, more marvellous than this which I have brought with me. I will be thy warrant for a speedy recovery." Thereupon mingling caresses with surgery, she salves the wondrous youth's gashes, and presently—

"He was both whole and sound,
And all so fierce for to fight.
So is the falcon to the flight."

And again, how grateful to follow Sir Eger at nightfall, after the unhappy issue of his contest with Sir Graysteel. We accompany him as he approaches the castle which stands convenient by the wayside. We see the beautiful young lady, clothed in a scarlet mantle embroidered with gold and pearls, issuing from her bower and slowly advancing to meet him. We hear her courteously proffer to him hospitable entertainment in the castle and the aid of the most cunning leeches in the whole country. We listen to Sir Eger declining this offer, but asking for rest and quiet for a single night. We see this charming lady, accompanied by her damsels, leading the mailed knight into her bower, and seating him on her own bed. Then baked fowls, spiced wine, and "bread of main" are set before him, and the hostess, with her own fair hands, washes the dust from his eyes, and with a refined courtesy takes no heed of Sir Eger's missing little finger, the sign that he has been vanquished. The glove was whole, but the finger was missing, and she might well know it was no accident; but perceiving that he "thought shame" of it, she did not ask either for his name, or from what country he came, or where he was going, but "eased him in all manere." She gives him a drink of great restorative virtue, and although,

"More weak and weary might no man be,
And dried of blood as any tree,"

he soon revives, and is able to "speak and answer make."

"She and her maids, those ladies three,
Of all my gear they spoilèd me;
Both of mine hauberk and mine actown,
Washed me syn, and laid me down.
With her own hands, white as milk,
She stopped my wounds full of silk,*
And syn laid me into a bed
That was with silken sheets spread."

* "Common lint," says Ellis, "most probably answers the purpose better: but while silk was a novelty, it was thought to possess many medical as well as other perfections."

The knight is now left to his repose, but the lady and her maidens assiduously watch him during the night, "amusing themselves with songs of love, till the notes of the birds from the neighbouring arbour warned them of the approach of day. The lady then brings to him in a horn, a medicated drink of a green colour, which was so potent that his pains immediately vanished. She again dressed all his wounds, drew from her wardrobe a silken shift, which she put on over his shirt, and then braced on his armour, the weight and pressure of which no longer distressed him."*

Who that is sick does not envy Sir Guy or Sir Eger his fair doctor?

The acquirements of some of these lady-physicians surpassed even those of the highest class of modern graduates in medicine, and were immeasurably beyond the most recent suggestions of the Medical Council for the better education of students.

Thus Felice who played such cruel pranks with poor Guy of Warwick's affections, and who is described as being gentle and demure as a ger-falcon, and courteous, free, and wise, was learned in the seven arts, "withouten miss." She was familiar with astronomy, and practised in arithmetic and geometry. "Of sophistry she was also witty," as well as of rhetoric; and she was a skilled musician, while in her knowledge of science none could equal her. All this learning was simply accessory to and for the purpose of furthering her proficiency in medicine.

Again, as another example, there is the rich lady, who, as we learn from the tale of "Les deux Amants," in Marie's lays, resided and had studied medicine at Salerno for *thirty* years. In that celebrated school she had acquired so profound a knowledge of *salves* and *drugs, herbs* and *roots, electuaries* and *drinks*, that she was enabled to concoct medicaments, second only in virtue to that great desideratum, the Elixir of Life.

Although, however, in the age of chivalry, some knowledge of medicine, and the art of dressing wounds, formed a portion of the accomplishments of all properly educated ladies, they were not on that account looked upon or recognised as regularly trained physicians. They were, in fact, amateurs who made up for the paucity of regular leeches; and their acquirements chiefly fitted them to play the part of clever nurses. They acted, moreover, mainly in the absence of, or in conjunction with, the proper leech. Thus Sir Eger's fair physician dresses his wounds and administers to him physic only after he has refused the aid of the ordinary leech; and in the legend of Chaitivel (Marie's Lays), the Queen of Beauty, in succouring the sole surviving knight who has fought

---

* See Ellis's *Early English Metrical Romances.*

for her favour, summons to her aid the three best physicians to be had, and, together with them, attends to his necessities.

One of the happiest indications of the actual relation of the lady-leech of the middle ages to the true leech is found in Chaucer's "Nonnes Preestes Tale." Chanticleer, it so happened, was disturbed one night by ill-dreams. Much depressed thereby he made moan to Dame Partlet, perched by his side. But she cried, Fie upon him! and reproved him sharply for being alarmed by a dream. "Han ye no manne's herte?" quoth she, "and hav a berde?"* and then she proceeds to expound to him how dreams arise from repletions and "oft of fume and of complexions," and concludes that his dream had arisen from superfluity of his "rede *colera* parde." She clenches her argument by citing Cato's advice not to heed dreams, and then proceeds to recommend her spouse Chanticleer what to do to obtain present relief, in the absence of an apothecary:—

"Now sire," quod she, "when we flee fro the bemes,
For Goddes love, as tak some laxatif:
Up peril of my soule and of my lif,
I conseil you the best, I wol not lie;
That both of coler, and of melancolie,
Ye purge you; *and for ye shal not tarie,*
*Though in this toun be non apotecarie,*
I shal myself two herbes techen you,
That shal be for your hele, or for your prow;†
And in our yerde, the herbes shall I finde,
The which han of hir propretee by kinde
To purgen you benethe, and eke above.
Sire, forgete not this for Goddes love,
Ye be ful colerike of complexion:
Ware that the sonne in his ascention
Ne finde you not replete of humours hote:
And if it do, I dare wel lay a grote,
That ye shal hav a fever tertiane,
Or elles an ague, that may be your bane.
A day or two ye shal have digestives
Of wormes, or ye take your laxatives,
Of laureole,‡ centauria, and fumetere,§—
Or elles of ellebor, that groweth there,
Of catapuce,|| or of gaitre-berries,¶
Or herbe we growing in our yard, that mery is:
Picke hem right as they grow, and ete hem in.
Beth mery, husbond, for your fader kin
Dredeth no dreme; I can say you no more."

The history of a remoter period records the names of several

* Beard. † Profit. ‡ Spurge-laurel.

§ Fumitory—"*purgat bilem et humores adustos.*"—Ray's Synopsis.

|| A species of spurge. ¶ Berries of the dog-wood tree, *Cornus fœmina.*

females who attained celebrity in the practice of medicine, chiefly in its relations to midwifery and the diseases of women. But as in the middle, so in earlier ages, we never find the names of those who had become thus celebrated, included among the true representatives of Æsculapius. The midwife of ancient Greece, in the time of Hippocrates, held no higher position than the midwife of a later period, before the practice of obstetric art passed almost entirely into the hands of men; and it is most probable that in difficult cases the regular physician was had recourse to. The story of Agnodice shows that even at the early period when she is presumed to have lived, the practice of midwifery was not entirely confined to women. A law had been passed in Athens, prohibiting females not only from acting as midwives, but from exercising the art of medicine in any form. This law had been called for in consequence of a prevalent custom existing among midwives and female dabblers in physic of procuring sterility and inducing abortion. Aspasia and Lais two of the most noted of the female physicians of antiquity, it may be remarked in passing, obtained their celebrity chiefly through their knowledge of drugs adapted to these nefarious ends. In the Oath found among the works of Hippocrates, the young physician is required to swear that he will not produce abortion.—"A notable instance," as Adams says, "how much our author (Hippocrates) was superior to his age in humanity as well as intelligence." Agnodice studied medicine secretly and practised midwifery in the disguise of a man; but being detected, she only escaped death by the powerful intercession of the principal women in Athens.

The practice of physic affected by the ladies of ancient days, would seem to have borne pretty much the same relation to the orthodox physic of that time, which the domestic medicine of the present day does to the orthodox physic of the present time. There were distinguished and historical Lady Bountifuls then, as there have been since, and the lady mesmeric, spiritualistic, and homœopathic practitioners of our own day, are the analogues of the witch-doctress of an earlier date, and the votress of Circe and other irregular healing goddesses of a still more remote period. Humanity was the same at the bottom in all ages.

Becker, in his Charicles, or illustrations of the social life of the ancient Greeks, represents the restless invalid as not content with seeking aid from the descendants of Æsculapius, but as having recourse also to enchantments, consulting the interpreters of dreams, placing expiations in the cross ways, and summoning aged women reputed to have the power of curing diseases by mysterious arts and magic ways, while whole days and nights were passed in the temple of Æsculapius.

Substitute "spiritualism" for enchantments, the clairvoyant for the interpreter of dreams, fees to charlatans for expiations placed in the cross ways, "mediums," or wise-women, a race not quite extinct, for the ancient witch, and medicated or Turkish baths for the temple of Æsculapius, and the picture is applicable to the present time.

That woman has never been admitted within the ranks of legitimate physic since it obtained recognition as a distinct science, notwithstanding her propensity to meddle with the healing art, may be looked upon as a pretty clearly established historical fact. The Asclepiadæ, the descendants of Æsculapius according to one version, the priesthood according to another, regarded the knowledge of medicine as a sacred trust, and bound themselves by oath (as recorded by Hippocrates, himself one of the Asclepiadæ) "to impart a knowledge of the Art to their own sons, and those of their teachers, and to disciples bound by a stipulation and oath according to the law of medicine, but to none others."

Why woman should have been excluded from the knowledge of medicine at its earliest origin as a separate profession, it is not difficult to surmise, without having recourse to the notion of a superstitious prejudice, or of the superior qualifications of man intellectually. The Father of Physic, in his admirable ideal portraiture of a true physician, "The Law," has laid down that whoever is to acquire a competent knowledge of medicine ought to be possessed of the following advantages:—a natural disposition; instruction; a favourable position for the study; early tuition; love of labour; and leisure.

From what we have already said it may be conceded unreservedly that woman has a natural aptitude for medicine; and it may also be granted that her intellectual capacity fully qualifies her for instruction, which instruction would follow if all other requirements were in conformity. But the real hitch commences in the question of her favourable position for the study. So long as maternity was (and is) looked upon as the crown of womanhood, the social and domestic duties which are a necessary consequence of marriage, would govern the early tuition of woman. This tuition would not only be unsuited to the acquisition of medicine, but it would also destroy that love of labour and deprive woman of that leisure which Hippocrates enumerates among the advantages to be possessed for that acquisition; since the leisure required is such as would be consistent with the proper study of medicine, and the love of labour such as would secure the right achievement of that study. But when, as in the case of woman, there was a primary and absorbing object of life in view, wife-hood, which rendered necessary a special training, it is evident that neither the leisure nor the labour could be accorded which are requisite for rightly mastering of physic.

It may reasonably be presumed that the ancients, in excluding woman from the study of medicine, reasoned somewhat after this fashion (as we reason now), if in fact they reasoned upon the matter at all. For it is just as probable, if not more so, that woman dropped quietly into the position which she holds with regard to medicine, by the natural action of her own feminine instincts. Be this as it may, however, those who of late years have advocated the admission of woman to the study of medicine, clearly see that, notwithstanding the hard verbal fashion in which they are accustomed to deal with poor male humanity, the chief obstacle is the domestic bias of woman herself and its consequences.

Thus Miss Dall, one of the most strenuous advocates of the fitness of woman for a more extended field of employment than is customarily yielded to her, and the object of whose life has been, to use her own words, "to inspire in woman a desire for *thorough training* to some special end, and a willingness to share the training of men both for specific and moral purposes," in a recent work,* makes known the history of a female physician, in the hope that it may do what her words never could, namely, "inspire women with faith to try their own experiments, give them a dignity which should refuse to look forward to marriage as an end, while it would lead them to accept it gladly as a providential help." But, alas! although generous men came forward to Miss Dall to carry out her plans for training women more largely to some business occupation, she was foiled by the women themselves. "The best printer in Boston said, 'I am willing to take women into my office at once, if you can find women who will submit to an apprenticeship like men.' On the same conditions, a distinguished chemist offered to take a class of women, and train them to be first-class apothecaries or scientific observers, as they might choose. To these offers there were no satisfactory responses. 'Yes,' said the would-be-printers, 'we will go into an office for six months; but, at that time, our oldest sisters will be married, and our mothers will want us at home.' 'An apprenticeship of six years!' exclaimed the young lady of a chemical turn; 'I should like to learn very much, so that I could be a chemist, *if I ever had to;* but poison myself for six years over those 'fumes,' not I.' "†

Undoubtedly the notion that the chief aim of woman's existence should be centred in a domestic life, has, on the one hand, narrowed for her the field of self-dependent labour, and, on the

---

* A Practical Illustration of "Woman's Right to Labour;" or a Letter from Marie E. Zakrzewska, M.D., late of Berlin, Prussia. Edited by Caroline H. Dall, Boston (U.S.) 1860.

† Op. cit., p. 2.

other, unfitted her too commonly for that labour. All honour, therefore, be to those energetic women who in view of the evils so frequently arising from these sources, are earnestly teaching the importance of training women to self-dependent exertion from girlhood. But we venture to suggest that the problem for solution is, in what manner this training can be effected without blunting the feminine qualities of woman, and not, as the ultra-teachers of this school, such as Miss Dall, for example, would have it, in what manner woman can be most nearly approximated in thoughts and habits to man. It is not so much a question, as these teachers will persist in arguing, of the intellectual or physical qualifications of woman for the different kinds of labour which have been monopolized by man, or any one of these, but it is a question of her moral fitness; for it is upon her moral and not upon her intellectual peculiarities that the chief characteristics of woman depend. Hence, in opening out new fields of labour to woman, the primary, the all-important question is, in what manner the preliminary and actual training required for them would affect her moral—her feminine qualities. And since upon these qualities depends the whole secret of domestic life, and all man's efforts are subsidiary to that life, and that upon its sacredness rests the integrity and progress of all true civilization, the question is by no means one which simply affects the condition of woman herself, or which can be dealt with by crude and vague notions of certain abstract "rights of labour" which woman may be presumed to possess.

There are probably several branches of labour, both manual and intellectual, which may still advantageously be thrown open to woman, but among these we do not rank medicine. We cannot conceive the possibility of woman studying, and above all, practising medicine, in any way worthy the name of study or practice, without to a greater or less extent unsexing herself. It would almost seem, in fact, as if this were done in anticipation by the proposition urged, and the attempts made, to pursue the study promiscuously with man; but we believe (at least we are wishful to suppose so) that both the proposition and the efforts made to carry it into effect, arise from sheer ignorance of the intimate requirements of medical art. But it may be said the experiment has been tried, and with success. We admit it, and the success neatly clenches the validity of our objection. Miss Dall, in the work referred to, publishes the life of Marie E. Zakrzewska, a Prussian midwife of unusual attainments, and who, by indomitable energy and perseverance, succeeded in obtaining admission to one of the American colleges, and graduating very honourably in medicine there. But at what a cost! She prides herself in ignoring and contemning all those feminine duties which have hitherto been looked upon as the chief of woman's claim to respect and honour, and she comes before us not as an ennobled woman, but as a feeble, feminine caricature of man! Miss Dall has given Dr. Marie E. Zakrzewska's life to the world as a triumphant illustration of women's capacity for masculine studies, and as an incentive to aspiring women. We strongly recommend all who are interested in this question to read the remarkable history. We are willing to admit that this lady may possess as a physician those high attainments which Miss Dall claims for her, but we look upon them as a miserable equivalent for the loss of those feminine traits which she makes so obtrusively, and it may be said boastfully, evident.* It is curious how ignorant, or wilfully heedless the ultra woman's rights advocates seem of the ways of the world. The ideal notion of the female physician comes to us recommended by the pleasant blending of the true feminine virtues of the woman with the qualifications of the physician. But in the example here presented to us, the female physician is divested of all those qualities which are so loveable in woman, and is represented solely as striving at the masculine standard of the physician. Is it so sure that the physician, who is but an imitation—a *quasi*-man, would be as well qualified to secure the confidence of the patient, whether male or female, as the physician who was the true man.

Still more instructive than Dr. Marie E. Zakrzewska's life is the fate which has befallen another American female physician, one of the best known and most distinguished of her class, and one who had won for herself the high respect of the medical authorities of Europe. She attempted to establish herself in general practice; yet although she had the sympathies of a large

---

* She revolts at the "common routine of domestic life" (p. 72), and has a private horror of knitting and sewing "under all conditions" (p. 156)—very commonplace avocations we admit, but commonplaces of that class without which the world would fare very badly. Did Doctor Marie Zakrzewska never reflect how the lack of aptitude for such commonplace occupations, in those women who were early trained to some special bread-getting occupation, was a very common source of domestic unhappiness, which alike demoralized husband, wife, and children? But Doctor Marie Zakrzewska's masculine propensities, it is but just to say, date from childhood. She tells us that she "joined the boys in all their sports; sliding and snow-balling with them in winter, and running and playing ball in summer. With them I was merry, frank, and self-possessed; while with the girls I was quiet, shy, and awkward. I never made friends with girls, or felt like approaching them." (p. 24.) As a girl, "she could not see why, at home, she should be bound to do housework when she wanted to read, while her brother, who wished to work, was compelled to study." (p. 39.) On her return home from school she began her apprenticeship in household duties, but as soon as the novelty wore off they "became highly burdensome," and were of course neglected, much to the discomposure and dissatisfaction of the household. (p. 43.) As a girl also, when a patient in the hospital, asking the physician under whose care she was, for books "about history," he gave her "two huge volumes—*The History of Midwifery* and *The History of Surgery*," which she read with interest!

circle of wealthy and influential friends, as well as physicians, she failed of patronage, and consequently of success. "She was found," says our authority,* "unable to meet the exigencies of the every-day duties of her profession, as every one practically familiar with the exacting nature of those duties would have foreseen. The storm, the cold, the night, the distance, were barriers which she could not overcome without assuming the habits, dress, and manners of the opposite sex. And often the disease which she encountered was of such a nature as to compel her either to unsex herself in regard to her instinctive habit of reticence and modesty, or preserve her feminine sensibilities by neglecting her professional duties. Subsequently she became the medical head of a private charity for the treatment of sick women, in which capacity her medical education is admirably adapted to develope and give efficiency to her natural tastes and her instincts, and thus render her life one of eminent usefulness."

It is significant that such success as Dr. Marie E. Zakrzewska had in practice was chiefly through her establishing an "Infirmary," in conjunction with Drs. Elizabeth and Emily Blackwell, and that she is now the head of a hospital in connexion with the New-England Female Medical College.

These illustrations sufficiently indicate the nature and the reality of the very grave objections to the study of medicine by females. It is not, we repeat it, any doubt of their intellectual or even physical capacity for the study and practice of medicine which governs our objections, but the impossibility of believing that the preliminary training requisite, the prescribed course of medical study itself, and the subsequent practice of the art in its integrity, can be consistent with woman retaining her true womanly habits and feelings.

If the question of female physicians were one which rested upon any real social or philanthropic need, it would at least appeal to our sympathies, but such is not the case. It arises incidentally, as we have seen, out of a fantastic doctrine of the "social rights" of women, which seeks to approximate her training and social status in every possible respect to that of man—aiming to make woman as masculine as practicable, and chafing that, after all, she must physically be woman still.† Consistently with this doctrine, we

---

* *American Medical Times*, July 23, 1861.

† Of the style of thought which passes for philanthropy among thinkers of this class, the following specimen may be taken from Doctor Marie Zakrzewska's life. She is writing of the experience of life she had gained when a girl by accompanying and aiding her mother, a Berlin midwife, in her professional visits and duties:—"One fact I learned, both at this time and afterwards, namely, that men always sympathise with fallen and wretched women, while women themselves are the first to raise and cast the stone at them. Why is this? Have not women as much feeling as men? Why, women are said to be made up entirely of feeling. How find its disciples, as if desirous of demonstrating their contempt for the hitherto received notions of female propriety, insisting upon the study of medicine as one peculiarly fitted for women, and also insisting upon their right to study, and the fitness of so studying, promiscuously with men. That is to say, we, who have been taught by experience that the education of males and females is best carried on apart the one from the other, are asked to admit that under circumstances which would usually be considered most obnoxious to the society of the sexes, a specific training in company is not only proper but advisable. Miss Dall protests* against the medical training of women in separate colleges, and hopes to see the conventional obstacles which have rendered this separation necessary, done away with in due time.†

It is curious to note, in further illustration of the sentiments of those ladies who belong to this school of thought, how subsidiary a position midwifery apparently holds in their estimation. To a woman of genuine feeling, with an inclination towards medicine, it might be supposed that the obstetric art would possess

---

does it happen, then, that women condemn where men pity? Do they do this in the consciousness of their own superior virtue? Ah, no! for many of the condemning are no better than the condemned. The reason is, that men know the world; that is, they know the obstacles in the path of life, and that they draw lines to exclude women from earning an honest livelihood, while they throw opportunities in their way to earn their bread by shame. All men are aware of this; therefore the good as well as the bad give pity to those who claim it. It is my honest and earnest conviction, that the reason that men are unwilling for women to enter upon public life or business life is, not so much the fear of competition, or the dread lest women should lose their gentleness and thus deprive society of this peculiar charm, as the fact that they are ashamed of the foulness of life which exists outside the house and home. The good man knows that it is difficult to purify it. The bad man does not wish to be disturbed in his prey upon society. If I could give to all women the least part of my experience, they would see that this is true; and would see, besides, that only faith in ourselves and in each other is needed to work reformation. Let woman enter fully into business, with its serious responsibilities and duties; let it be made as honourable and as profitable to her as to man; let her have an equal opportunity for earning competence and comfort, and we shall need no other purification of society. Men are no more depraved than women; or rather, the total depravity of mankind is a lie." (p. 45.)

* *Woman's Right to Labour.* Boston, 1860.

† Dr. Marie Zakrzewska thus writes of her association with the male students in the School of Midwifery, Berlin:—"My relation with these young men was of the pleasantest kind. They never seemed to think that I was not of their sex, but always treated me like one of themselves. I knew of their studies and their amusements; yes, even of their mischievous pranks that they were planning both for college and for social life. They often made me their confidante in their private affairs, and were more anxious for my approval or forgiveness than that of their relatives. I learned during this time how great is the friendly influence of a woman even upon fast living and licentious young men; and this has done more to convince me of the necessity that the two sexes should live together from infancy, than all the theories and arguments that are brought to convince the mass of this fact. As soon as it became known among the students that my youth was the new objection [to her being admitted to the hospital], they treated it in such a manner that the whole thing was transformed into a ridiculous bugbear, growing out of the imagination of *virtuous* opposers." (p. 69.)

unusual attractions. To meet and emulate upon his own ground, and finally, perhaps, dispossess the man-midwife, would be a natural object of ambition ; to ennoble the female practice of midwifery, and open it out to a more highly educated class of women than have commonly practised it hitherto, would be a still loftier object. Woman has already attained the highest scientific distinction in midwifery (witness Mesdames Boivin and La Chapelle), and it would be hard to say that there is not a wide and noble field still open to her in its cultivation, if she were so disposed. The field, moreover, is a legitimate one, and the obstacles in the way, although sufficiently great, are perhaps not more than would suffice for a stimulus to one whose heart was in the matter and who was wishful to set a good example to others. But, singularly enough, feelings of this kind seem to exercise but a very slight influence over these ladies.

Dr. Marie E. Zakrzewska was originally educated as a midwife, and her ability was such that, when only twenty-two years of age, she received the appointment of chief midwife in the Royal Hospital at Berlin. She had also been legally appointed assistant to the professor in the School of Midwives, Dr. Schmidt, having displayed high qualifications as a teacher, and with the avowed intention on his part that she should become his successor—a hope, however, cut short by his sudden death on the day when she was to have commenced her duties as assistant professor. Internal dissensions in the hospital, by which she was victimized, caused her, soon after Dr. Schmidt's death, to resign her post. She did not lack friends in Berlin ; and not only was there the prospect before her of establishing a practice, but it had been suggested, and had met the approval of the physicians who befriended her, that a private hospital should be formed and put under her charge. She had been playfully termed La Chapelle the Second, and there was little reason to doubt that she would have justified the appellation, and that she might probably look forward to obtaining European celebrity. It may be readily imagined how a success of this kind could have been made available to opening the practice of midwifery more fully to females ; but to justify woman's right to labour in this fashion does not seem to have occurred to her.* She set the advantages she possessed aside, and emigrated to America, seduced by the prospect of being able there to graduate fully in medicine,† and submitting to a bitter

* The American Secretary of Legation at Berlin (1853), T. S. Fay, in a certificate attesting Miss Zakrzewska's claims, states that the acknowledged excellence of the midwives of that city threatens to monopolize the obstetric art.

† "I had come here for a purpose," she writes, speaking of her first arrival in America, "to carry out the plan which a despotic government and its servile agents had prevented me from doing in my native city. I had to show to those men who had opposed me so strongly because I was a woman, that in this land of liberty, struggle with poverty to attain her end—a struggle which, however, chips off the veneer of misdirected thought, and enables us to see the true woman. For while earning her bread by woolwork in New York, she contrives effectually to aid some of her own distressed or lost sex, giving an asylum to several, even in her own house, and teaching how woman can truly ennoble herself.

Neither in the origin, nor the development, nor the results (so far as these are yet apparent), of the question of female physicians, has there been any sufficient reason advanced why our schools of medicine and medical diplomas or licences should be laid open to female students and candidates; and there are certain manifest and most weighty reasons arising from the intrinsic nature of medicine, why this course should not be adopted. It does not follow that these reasons are mere conventional prejudices, because certain persons are to be found with whom they have no influence, and that in America chartered medical schools have been established for females exclusively, and also for males and females, while public opinion there sets strongly in favour of the medical education of females. The establishment of these schools merely shows that there is a class of people with whom certain old-fashioned, but as yet very vital and fundamental notions of feminine duty and propriety have become greatly modified, and that it is prepared to test the correctness of its views by experience. Hitherto the results have not been such as to remove, but rather to confirm, the objections we have noted.

We can conceive that many plausible reasons might be advanced for training female physicians to practise in midwifery, and the diseases of women and children; but, as the question has never been broached in this form, we need not dwell upon them.

Woman always has and ever must play an important part in the art of physic, a part which has been assigned to her for ages, and which offers the widest scope for the exercise of her highest intellectual and moral attainments. Hippocrates, in the Oath we have previously referred to, swears by "Apollo the physician and Æsculapius, Health and All-Heal." Health (Hygeia), and All-Heal (Panacea), and Brightness (Ægle), and Recovery (Jaso), were the four daughters of Æsculapius. To his two sons, Podalirius and Machaon, Æsculapius bequeathed the science and art of healing, to his four daughters those virtues without which

---

equality, and fraternity, I could maintain that position which they would not permit me at home. My talents were in an unusual direction. I was a physician, and as such, had moved in the most select circles of Berlin. Even my enemies had been forced to give me the highest testimonials [testimonials as a midwife are alone specified], and these were the only treasures I brought to this country."—(p. 97.)

all physic must prove of no avail. Hygeia is the deified representative of the many feminine virtues which in maternal and domestic life are requisite to secure the health of childhood and youth and to promote a vigorous maturity; Ægle watches over the perfection of Hygeia's cares; Panacea is the tender nurse whose affectionate kindness in sickness soothes the fever-tossed or pain-racked sufferer, and whose watchful care fosters the all-healing powers of nature, and facilitates and perfects the feeble efforts of art; Jaso cherishes Panacea's completed work, and renovates the convalescent's exhausted, but now no longer disease-oppressed powers.

The deified daughters of Æsculapius, in fact, represent with tolerable accuracy the notions which have been customarily held from remote to the most recent times of the proper relationship of Woman to medicine, and to these notions we hold. The midwife is no true exception; for labour is a natural process, and her relation to it was, and is, simply in its natural phases, and not in those unfortunate modifications of the process, which from the beginning of medicine rendered the aid of the physician occasionally necessary, and which ultimately led to midwifery becoming a branch of medical science and a part of his duties.

---

